# Author's reply

**Published:** 2010

**Authors:** Samar K Basak, Arup Bhaumik, Ayan Mohanta, Prashant Singhal

**Affiliations:** Disha Eye Hospitals and Research Centre, Barrackpore, West Bengal, Kolkata - 700 120, India

Dear Editor,

The authors wish to thank Lakhtakia *et al.*[1] for their interest in our article and for their comments.[2]

In Bengal, the flower vendors are predominantly male, and direct worshipping rites in the temple are performed by male Brahmin pundits, called *purohits* and hence there was male preponderance.

We do not have an explanation as to why there was secondary glaucoma in seven (24.1%) cases. We had just analyzed the reports retrospectively. As we routinely performed noncontact tonometry in all our patients attending the outpatient department, we had picked up those intraocular pressure rises in some patients.

The references that the author has given as Reference Nos. 2, 3 and 4 in his letter[1] are not published in peer review-indexed journals. That is why they were not included.

Reference No. 5 was published in April, 2008 in Cornea Journal. We had submitted our manuscript in July 2007. Therefore, there was no scope of mentioning this reference in our report.
